# Prognostic value of NT-proBNP in patients with chronic coronary syndrome and normal left ventricular systolic function according to glucose status: a prospective cohort study

**DOI:** 10.1186/s12933-021-01271-0

**Published:** 2021-04-22

**Authors:** Hui-Hui Liu, Ye-Xuan Cao, Jing-Lu Jin, Yuan-Lin Guo, Cheng-Gang Zhu, Na-Qiong Wu, Ying Gao, Yan Zhang, Rui-Xia Xu, Qian Dong, Jian-Jun Li

**Affiliations:** State Key Laboratory of Cardiovascular Disease, FuWai Hospital, National Center for Cardiovascular Diseases, National Clinical Research Center for Cardiovascular Diseases, Chinese Academy of Medical Sciences and Peking Union Medical College, No. 167 BeiLiShi Road, XiCheng District, 100037 Beijing, China

**Keywords:** N-terminal pro‐brain natriuretic peptide, Chronic coronary syndrome, Cardiovascular outcomes, Prediabetes, Diabetes mellitus, Risk factor

## Abstract

**Background:**

The prognostic value of N-terminal pro-brain natriuretic peptide (NT-proBNP) in patients with coronary artery disease (CAD) with different glucose status has not been established. This study sought to evaluate the significance of NT-proBNP in predicting major adverse cardiovascular events (MACEs) in patients with chronic coronary syndrome (CCS) and normal left-ventricular systolic function (LVSF) according to different glucose status, especially in those with abnormal glucose metabolism.

**Methods:**

A total of 8062 patients with CCS and normal LVSF were consecutively enrolled in this prospective study. Baseline plasma NT-proBNP levels were measured. The follow-up data of all patients were collected. Kaplan-Meier and Cox regression analyses were used to assess the risk of MACEs according to NT-proBNP tertiles stratified by glucose status.

**Results:**

Over an average follow-up of 59.13 ± 18.23 months, 569 patients (7.1 %) suffered from MACEs, including cardiovascular death, non-fatal myocardial infarction, and non-fatal stroke. Kaplan-Meier analysis showed that high NT-proBNP levels had a significant association with MACEs in subjects with prediabetes mellitus (pre-DM) or DM, but not in patients with normoglycemia. Multivariate Cox regression analysis revealed that NT-proBNP remained an independent predictor of MACEs in patients with pre-DM [hazard ratio (HR): 2.56, 95% confidence interval (CI): 1.34–4.91] or DM (HR: 2.34, 95% CI: 1.32–4.16). Moreover, adding NT-proBNP to the original Cox model including traditional risk factors significantly increased the C-statistic by 0.035 in pre-DM and DM, respectively.

**Conclusions:**

The present study indicated that NT-proBNP could well predict worse outcomes in dysglycemic patients with CCS and normal LVSF, suggesting that NT-proBNP may help with risk stratification in this population.

**Supplementary Information:**

The online version contains supplementary material available at 10.1186/s12933-021-01271-0.

## Background

B-type natriuretic peptide (BNP) and N-terminal pro-BNP (NT-proBNP) are synthesized in cardiomyocytes and released into circulation in response to volume overload and cardiac stress, and thereby mirror a fundamental pathobiological mechanism of cardiovascular disease (CVD) [1]. Currently, NT-proBNP has become the focus of cardiac risk markers [2]. It has been well established that NT-proBNP is an excellent biomarker of heart failure (HF) independent of the underlying heart disease [3] and its plasma concentrations are predictive of worse outcomes in these patients [4–6]. Interestingly, more uses for this biomarker have been discovered in recent years. For example, Von Jeinsen et al. [7] found that there was a strong, positive association of NT-proBNP with fatty-acid binding protein 4 (FABP4) levels, while FABP4 may have a dose-dependent association with cardiac remodeling. A recently published study showed that NT-proBNP was a useful biomarker of cardiac conditions in patients undergoing left ventricular assist device implantation [8]. Moreover, NT-proBNP was also suggested to be a strong predictor for mortality and cardiovascular events (CVEs) in the general population [9–11], patients with diabetes mellitus (DM) [2, 4, 12–15], acute coronary syndrome (ACS) [14], and chronic coronary syndrome (CCS) [16, 17]. Further exploration of the application value of NT-proBNP in wider populations has become a hot topic in cardiovascular field.

The recently published 2019 ESC guidelines specifically focus on the risk assessment, prevention, and management of prediabetes mellitus (pre-DM), DM, and CVD [18]. As well known, DM in general confers a two-fold higher risk of CVEs independent of other risk factors [18]. Meanwhile, pre-DM is an intermediate metabolic state between normoglycemia and DM with an increasing morbidity rate as the growing obesity epidemic [19]. According to the American Diabetes Association (ADA) criteria [20], the prevalence of pre-DM in adults was up to 36.2% in the US and 50.1% in China [21]. This population are at high risk for DM. The predisposition of pre-DM to DM makes it a potential risk factor for CVD and arouses great interest in cardiovascular medicine [21, 22]. Thus, identifying a subpopulation of patients with dysglycemia who are at absolutely high cardiovascular risk is of great significance, since they would probably benefit more from preventive therapeutic strategies. Undoubtedly, intensive study of patients with abnormal glucose metabolism is imperative.

We hypothesized that NT-proBNP might be a useful predictor for worse outcomes in patients with pre-DM or DM combined with CCS as well. Hence, in this study, we sought to investigate the association of NT-proBNP with long-term major adverse CVEs (MACEs) in patients with CCS and normal left-ventricular systolic function (LVSF) according to three status of glucose metabolism, especially in those with pre-DM or DM.

## Methods

### Study design and population

**Fig. 1:**
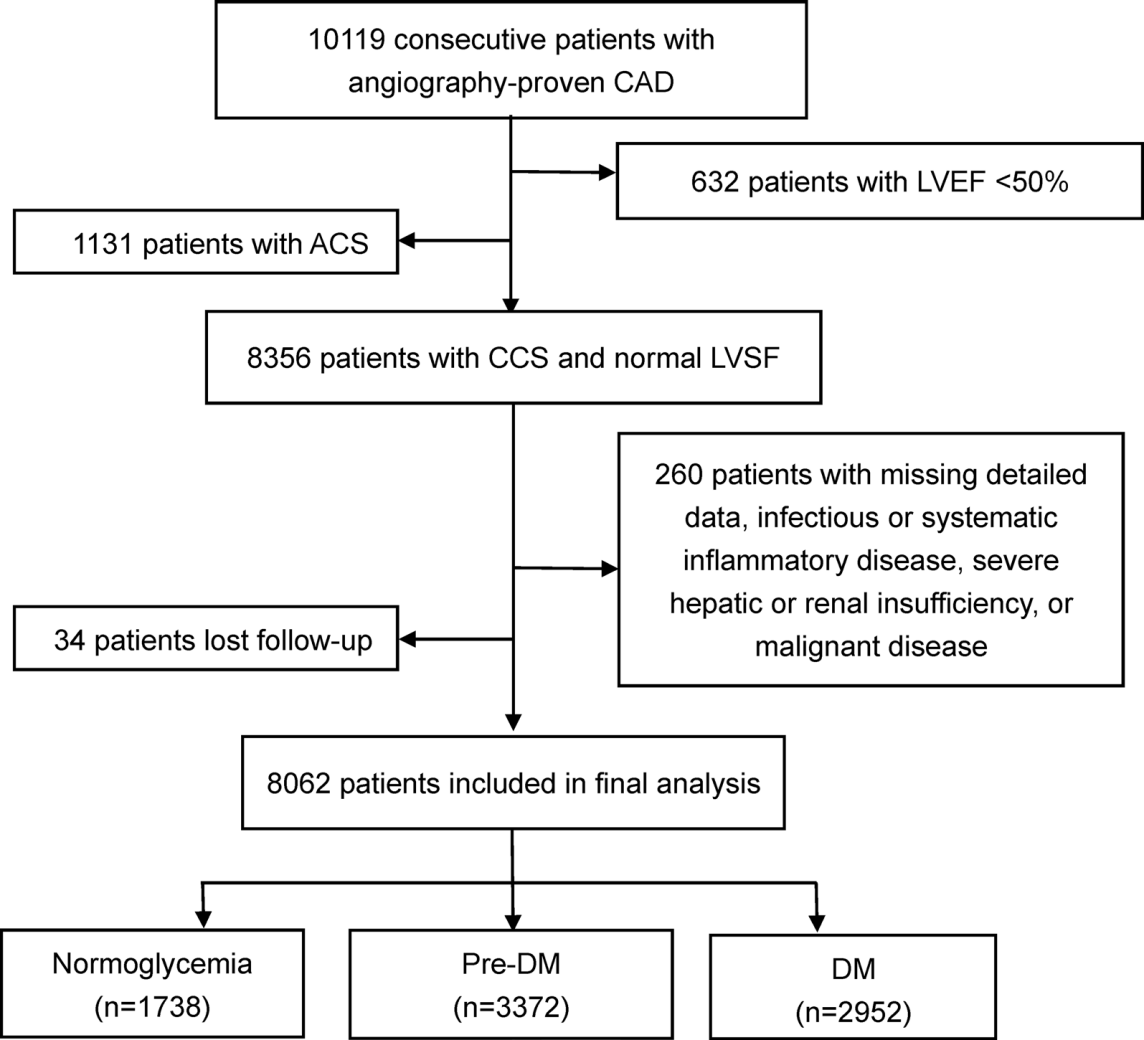
Flowchart illustrating study population. *ACS* acute coronary syndrome, *CAD* coronary artery disease, *CCS* chronic coronary syndrome, *DM* diabetes mellitus, *LVEF* left ventricular ejection fraction, *LVSF* left-ventricular systolic function, *Pre-DM* prediabetes mellitus

From March 2011 to December 2017, a total of 10,119 consecutive patients were diagnosed with CAD according to coronary angiography. Excluding 632 patients with left ventricular ejection fraction (LVEF) < 50% and 1131 patients with ACS [based on elevated myocardial enzyme levels including cardiac troponin I (cTnI), creatine kinase (CK), and CK-MB, typical electrocardiogram changes, and medical history], 8356 subjects with CCS and normal LVSF were eligible. Subsequently, 260 patients were excluded due to missing detailed laboratory data, infectious or systematic inflammatory disease, severe hepatic or renal insufficiency, or malignant disease, and 34 patients were lost to follow-up. Finally, 8062 patients were included in the analysis (Fig. 1).

### Biochemical analysis

Blood samples were taken by direct venipuncture from each patient after at least 12-h fasting in the morning. The samples were collected into EDTA-anticoagulant tubes and centrifuged to produce plasma. Plasma NT-proBNP concentration was measured with an electrochemiluminescence immunoassay (ECLIA) method (NT-proBNP, Roche, Germany) by a Roche modular analytics E170 immunoassay analyzer. Fasting blood glucose (FPG) was determined by enzymatic hexokinase method, while glycosylated hemoglobin (HbA1c) was measured using Tosoh Automated Glycohemoglobin Analyzer (HLC-723G8, Tokyo, Japan). Lipid profiles including total cholesterol, triglyceride, low-density lipoprotein cholesterol (LDL-C) and high-density lipoprotein cholesterol (HDL-C) were measured using an automatic biochemistry analyzer (Hitachi 7150, Tokyo, Japan) and enzymatic assay. The concentrations of high-sensitivity C-reactive protein (hsCRP) were determined using immunoturbidimetry (Beckmann Assay 360, Bera, CA, USA).

### Clinical assessment

On admission, demographic data and medical history including cardiovascular risk factors were collected from each patient. Glucose metabolism status was categorized according to the ADA 2010 criteria [20]: DM was diagnosed according to FPG ≥ 7.0 mmol/L, the 2-h plasma glucose of the oral glucose tolerance test ≥ 11.1mmol/L, HbA1c level ≥ 6.5%, or currently using hypoglycaemic drugs or insulin. Pre-DM was diagnosed when participants who had no self-reported DM or hypoglycaemic therapies but with a FPG range from 5.6 to 6.9 mmol/L, 2-h glucose range from 7.8 to 11.0 mmol/L, or HbA1c level range from 5.7 to 6.4%, while subjects without DM or pre-DM were defined as normoglycemia. Hypertension was defined by a self-reported hypertension, currently taking antihypertensive drugs, or recorded systolic blood pressure ≥ 140 mmHg and/or diastolic blood pressure ≥ 90 mmHg for three or more consecutive times. Current smoking was ascertained as regular smoking within the previous 12 months.

### Follow‐up

Patients were followed-up at 6 months’ intervals by means of interviewing directly or telephone communications by well-trained nurses or cardiologists who were blinded to the aim of the study. All events were carefully checked and verified by three experienced clinical physicians. The MACEs included cardiovascular death, non-fatal myocardial infarction (MI), and non-fatal stroke. Cardiovascular death was diagnosed as death mainly caused by acute MI, malignant arrhythmia, HF, or other structural or functional cardiac diseases. Non-fatal MI was defined according to positive cardiac troponins along with typical chest pain or typical electrocardiogram serial changes. Stroke was defined by persistent neurological dysfunction with documentation of acute cerebral infarction on computed tomography and/or magnetic resonance imaging.

### Statistical analysis

Continuous variables are expressed as mean ± SD or median (Q1–Q3 quartiles) as appropriate. The Kolmogorov-Smirnov test was used to test the distribution pattern. The differences between groups were determined using the Student's t-test, analysis of variance or nonparametric test where appropriate. Categorical variables are presented as number (percentage) and analyzed by χ^2^-test or Fisher’s exact test. The event-free survival rates among groups were estimated by the Kaplan–Meier analysis and compared by the log-rank test. Cox proportional hazard models were used to calculate the hazard ratios (HRs) and 95% confidence intervals (CIs). The multivariable model was adjusted for the following covariates in an all-enter way: age, sex, hypertension, current smoking, systolic blood pressure (SBP), creatinine, LDL-C, hsCRP, and baseline statin use. The associations between NT-proBNP and outcomes were examined using this biomarker in a categorical way and as a continuous variable, according to glucose status. To evaluate whether adding NT-proBNP to the original model could improve the ability for predicting MACEs, we calculated Harrell’s C-statistic. Two-tailed p-values < 0.05 were considered statistically significant. The statistical analyses were performed with SPSS version 24.0 software (SPSS Inc., Chicago, IL, USA) and R language version 3.5.2 (Feather Spray).

## Results

### Baseline characteristics

Among the subjects, 41.8% were defined as pre-DM, 36.6% had DM, and the rest 21.6% were with normoglycemia (Fig. 1). The baseline characteristics of the study population stratified by glucose status are shown in Table 1. Patients with pre-DM or DM were more likely to have hypertension, percutaneous coronary intervention (PCI), and coronary artery bypass grafting (CABG) histories, and less likely to be males and current smokers. The age, body mass index, SBP, NT-proBNP, FPG, HbA1c, triglyceride, and hsCRP levels were positively associated with the diabetes status from normoglycemia to DM. Meanwhile, individuals with pre-DM had higher total cholesterol and LDL-C levels than participants with normoglycemia or DM. DM group had slightly lower HDL-C and LVEF levels compared with the other two groups. Additionally, the use of angiotensin converting enzyme inhibitors/angiotensin receptor blockers, β-blockers, and calcium channel blockers at baseline and follow-up were more common in patients with dysglycemia than in subjects with normoglycemia.

**Table 1:** Characteristics of the study participants according to glucose status at baseline

Variable	Overall (n = 8062)	Normoglycemia (n = 1738)	Pre-DM (n = 3372)	DM (n = 2952)	*p* value
Age, years	57.8 ± 10.3	54.4 ± 10.8	58.4 ± 9.8	59.1 ± 10.1	< 0.001
Male, n (%)	5784 (71.7)	1331 (76.6)	2377 (70.5)	2076 (70.3)	< 0.001
Hypertension, n (%)	5074 (62.9)	972 (55.9)	2006 (59.5)	2096 (71.0)	< 0.001
Current smokers, n (%)	3381 (41.9)	789 (45.4)	1396 (41.4)	1196 (40.5)	0.004
Family history of CAD, n (%)	1135 (14.1)	267 (15.4)	472 (14.0)	396 (13.4)	0.166
Prior PCI, n (%)	2143 (26.6)	401 (23.1)	873 (25.9)	869 (29.4)	< 0.001
Prior CABG, n (%)	206 (2.6)	21 (1.2)	84 (2.5)	101 (3.4)	< 0.001
Prior MI, n (%)	2243 (27.8)	488 (28.1)	934 (27.7)	821 (27.8)	0.945
Prior stroke, n (%)	295 (3.7)	56 (3.2)	118 (3.5)	121 (4.1)	0.252
BMI, kg/m^2^	25.88 ± 3.17	25.43 ± 3.12	25.70 ± 3.18	26.35 ± 3.13	< 0.001
SBP, mmHg	127 ± 17	125 ± 17	126 ± 17	129 ± 17	< 0.001
DBP, mmHg	78 ± 11	78 ± 11	77 ± 11	78 ± 11	0.013
LVEF, %	64.11 ± 6.86	64.39 ± 6.68	64.29 ± 6.93	63.75 ± 6.87	0.001
NT-proBNP, pg/mL	323.7 (59.6–571.0)	163.0 (46.9-489.7)	352.6 (64.1-590.3)	368.0 (67.1-602.6)	< 0.001
FPG, mmol/L	5.86 ± 1.77	4.81 ± 0.44	5.25 ± 0.63	7.18 ± 2.26	< 0.001
HbA1c, %	6.32 ± 1.10	5.37 ± 0.24	5.93 ± 0.27	7.32 ± 1.21	< 0.001
TC, mmol/L	4.13 ± 1.16	4.06 ± 1.15	4.18 ± 1.14	4.10 ± 1.18	< 0.001
HDL-C, mmol/L	1.06 ± 0.29	1.06 ± 0.30	1.08 ± 0.29	1.03 ± 0.28	< 0.001
LDL-C, mmol/L	2.50 ± 1.00	2.47 ± 1.06	2.54 ± 0.95	2.46 ± 1.01	0.002
TG, mmol/L	1.49 (1.10–2.08)	1.40 (1.01–1.98)	1.49 (1.10–2.03)	1.56 (1.17–2.20)	< 0.001
Creatinine, umol/L	77.79 ± 18.20	77.99 ± 15.76	77.44 ± 18.37	78.08 ± 19.32	0.328
HsCRP, mg/L	1.35 (0.74–2.80)	1.08 (0.64–2.19)	1.36 (0.74–2.87)	1.52 (0.83–3.13)	< 0.001
Baseline medications					
Aspirin, n (%)	6053 (75.1)	1296 (74.6)	2525 (74.9)	2232 (75.6)	0.691
Statins, n (%)	6195 (76.8)	1317 (75.8)	2590 (76.8)	2288 (77.5)	0.518
ACEI/ARB, n (%)	1728 (21.4)	315 (18.1)	708 (21.0)	705 (23.9)	< 0.001
β-blockers, n (%)	3364 (41.7)	624 (35.9)	1447 (42.9)	1293 (43.8)	< 0.001
CCB, n (%)	1597 (19.8)	311 (17.9)	658 (19.5)	628 (21.3)	0.070
Follow-up medications					
Aspirin, n (%)	7991 (99.1)	1722 (99.1)	3338 (99.0)	2931 (99.3)	0.451
Statins, n (%)	7807 (96.8)	1679 (96.6)	3274 (97.1)	2854 (96.7)	0.531
ACEI/ARB, n (%)	3822 (47.4)	744 (42.8)	1528 (45.3)	1550 (52.5)	< 0.001
β-blockers, n (%)	6275 (77.8)	1283 (73.8)	2607 (77.3)	2385 (80.8)	< 0.001
CCB, n(%)	3119 (38.7)	603 (34.7)	1285 (38.1)	1231 (41.7)	< 0.001

Continuous values are summarized as mean ± SD, median (interquartile range) and categorical variables as percentage

*ACEI* angiotensin converting enzyme inhibitors, *ARB* angiotensin receptor blockers, *BMI* body mass index, *CCB* calcium channel blockers, *CABG* coronary artery bypass grafting, *DM* diabetes mellitus, *DBP* diastolic blood pressure, *FPG* fasting plasma glucose, *HbA1c* glycosylated hemoglobin, *HDL-C* high-density lipoprotein cholesterol, *HsCRP* high sensitivity C-reactive protein, *LVEF* left ventricular ejection fraction, *LDL-C* low-density lipoprotein cholesterol, *MI* myocardial infarction, *NT-proBNP* N-terminal pro-B-type natriuretic peptide, *PCI* percutaneous coronary intervention, *SBP* systolic blood pressure, *TC* total cholesterol, *TG* triglyceride

### NT-proBNP and MACEs

Among the patients with CCS, 569 experienced new-onset MACEs (208 cardiovascular deaths, 122 non-fatal MIs, and 239 strokes) after an average follow-up of 59.13 ± 18.23 months, with an incidence rate per 1000 person-years of 9.8 (95% CI: 5.1–14.5) in normoglycemia, 13.8 (95% CI: 9.9–17.7) in pre-DM, and 17.8 (95% CI: 13.1–22.5) in DM, respectively. Obviously, patients with pre-DM or DM had a significantly higher incidence of MACES compared with those with normoglycemia (Additional file 1: Figure S1). The demographic and biochemical characteristics with respect to incident MACEs are summarized in Additional file 1: Table S1. Subjects who suffered from MACEs had significantly higher NT-proBNP (556.2 vs. 301.5 pg/mL, *p* < 0.001), SBP, HbA1c, creatinine, and hsCRP levels than those without events. In addition, patients with MACEs were slightly older and presented a higher prevalence of hypertension, DM, prior CABG and MI, compared to those without MACEs.

**Fig. 2:**
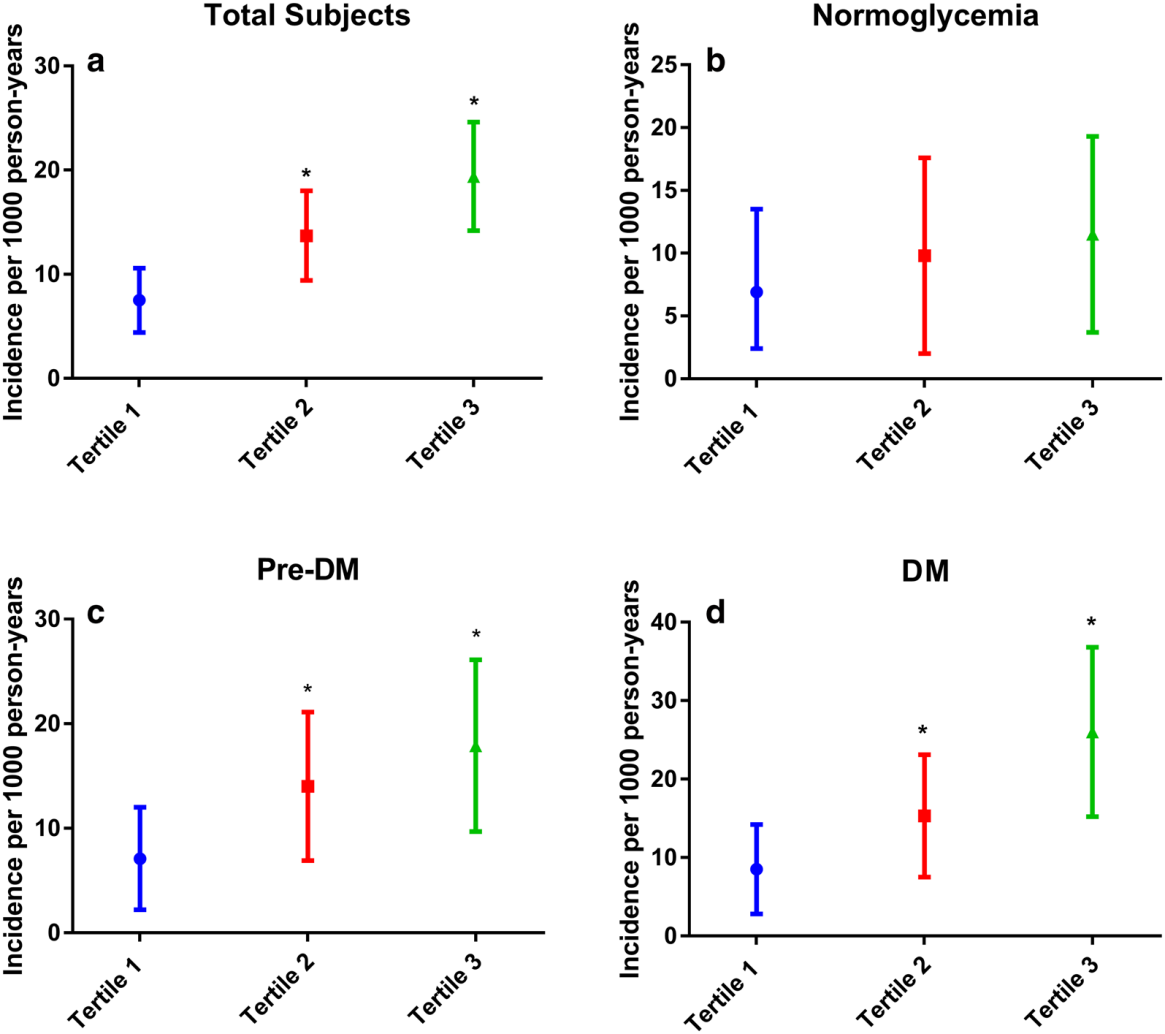
The incidence rate of MACEs across NT-proBNP tertiles stratified by glucose status. **a** Total subjects. **b** Normoglycemia. **c** Pre-DM. **d** DM. *DM* diabetes mellitus, *MACE* major adverse cardiovascular event, *NT-proBNP* N-terminal pro-B-type natriuretic peptide, *Pre-DM* prediabetes mellitus. * *p* < 0.0167 compared with Tertile 1 group

**Fig. 3:**
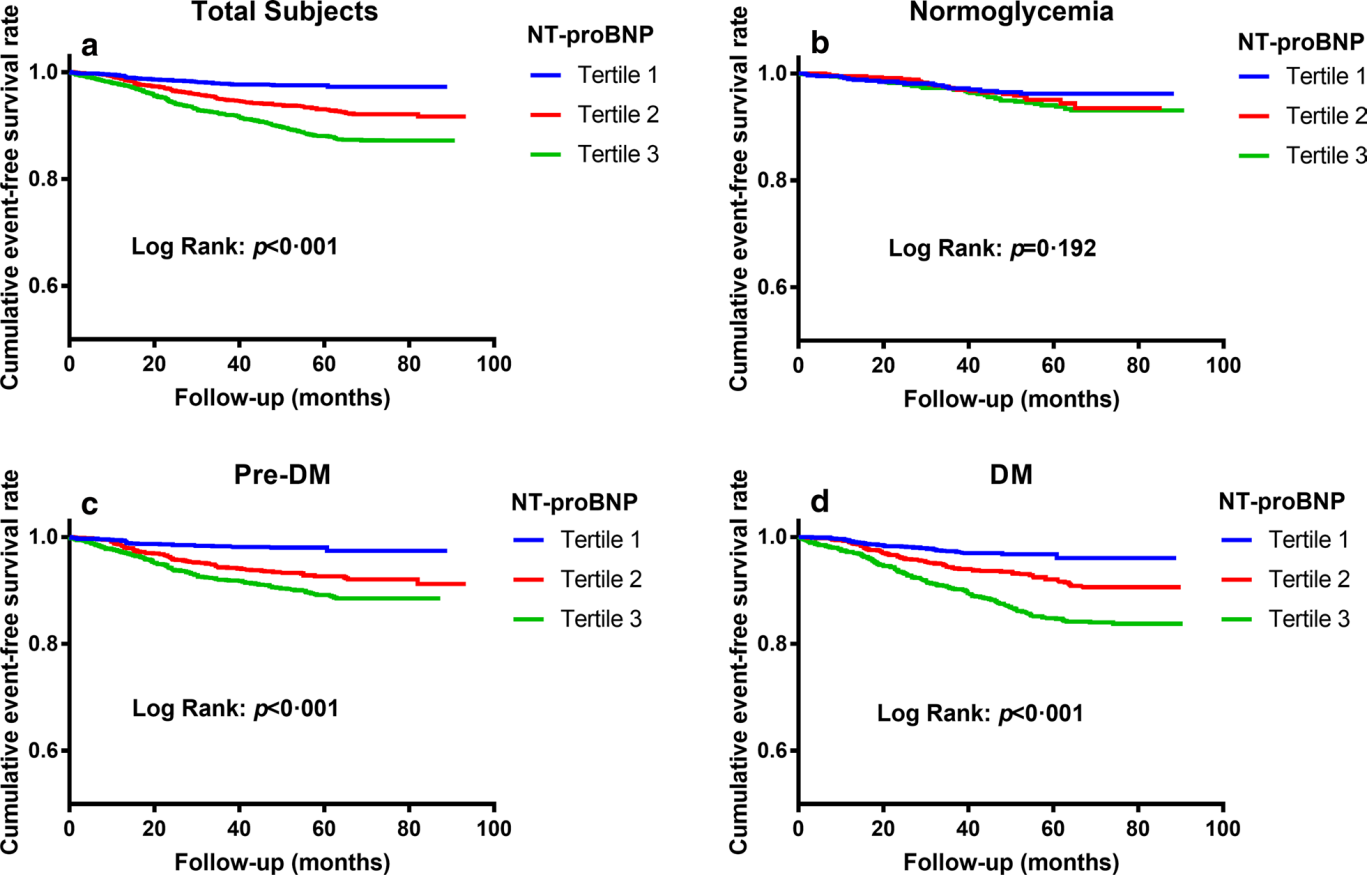
The cumulative event-free survival analysis across NT-proBNP tertiles stratified by glucose status. **a** Total subjects. **b** Normoglycemia. **c** Pre-DM. **d** DM. *DM* diabetes mellitus, *NT-proBNP* N-terminal pro-B-type natriuretic peptide, *Pre-DM* prediabetes mellitus

As shown in Fig. 2a, for all patients, compared with those in tertile 1 of NT-proBNP, patients in tertile 2 or tertile 3 had significantly higher levels of incidence rate of MACEs. In subgroup analyses according to glucose status, we observed similar results in patients with pre-DM and DM (Fig. 2c, d), but not in those with normoglycemia (Fig. 2b). The further Kaplan-Meier analysis also showed that in the overall population, patients in higher two tertiles of NT-proBNP had significantly lower cumulative event-free survival rates compared with those in tertile 1 (Fig. 3a), so did in the subgroup of pre-DM or DM (Fig. 3c, d). However, there was no significant difference of event-free survival rate among three tertiles in the normoglycemia group (Fig. 3b).

In Cox regression models incorporating NT-proBNP as tertiles or as a continuous variable, the elevated risk of MACEs associated with increased levels of NT-proBNP persisted in patients with pre-DM (adjusted HR 2.39, 95% CI 1.25–4.55 for tertile 2 vs. tertile 1; adjusted HR 2.56, 95% CI 1.34–4.91 for tertile 3 vs. tertile 1) or DM (adjusted HR 1.51, 95% CI 1.04–2.20 for tertile 2 vs. tertile 1; adjusted HR 2.34, 95% CI 1.32–4.16 for tertile 3 vs. tertile 1). Per 1-SD increase of log-transformed NT-proBNP was associated with a 61% increase of the risk of MACEs in patients with CCS and pre-DM, while a 69% increase in those with CCS combined with DM (Table 2). In addition, the multivariate Cox regression analyses showed that NT-proBNP was the strongest marker for predicting MACEs in dysglycemic patients with CCS (Additional file 1: Table S2).

**Table 2:** Cox regression analyses of NT-proBNP for predicting MACEs according to glucose status at baseline

Category	Crude HR (95% CI)	Adjusted HR (95% CI)
Overall^a^		
LgNT-proBNP (per 1-SD)	2.82 (2.21–3.60)^‡^	1.61 (1.35–1.91)^‡^
Tertile 1 (< 92.5 pg/mL)	1.00 (reference)	1.00 (reference)
Tertile 2 (92.5-491.1 pg/mL)	2.10 (1.45–3.03)^‡^	1.89 (1.24–2.86)^†^
Tertile 3 (> 491.1 pg/mL)	3.24 (2.29–4.60)^‡^	2.65 (1.77–3.98)^‡^
Normoglycemia		
LgNT-proBNP (per 1-SD)	1.32 (1.05–1.66)*	1.12 (0.76–1.65)
Tertile 1 (< 65.5 pg/mL)	1.00 (reference)	1.00 (reference)
Tertile 2 (65.5-405.8 pg/mL)	1.37 (0.77–2.44)	1.31 (0.73–2.35)
Tertile 3 (> 405.8 pg/mL)	1.65 (0.96–2.85)	1.50 (0.83–2.71)
Pre-DM		
LgNT-proBNP (per 1-SD)	1.96 (1.54–2.50)^‡^	1.61 (1.23–2.12)^‡^
Tertile 1 (< 101.8 pg/mL)	1.00 (reference)	1.00 (reference)
Tertile 2 (101.8–507.0 pg/mL)	2.59 (1.45–4.64)*	2.39 (1.25–4.55)^†^
Tertile 3 (> 507.0 pg/mL)	3.64 (2.07–6.39)^‡^	2.56 (1.34–4.91)^†^
DM		
LgNT-proBNP (per 1-SD)	1.92 (1.52–2.42)^‡^	1.69 (1.30–2.21)^‡^
Tertile 1 (< 110.6 pg/mL)	1.00 (reference)	1.00 (reference)
Tertile 2 (110.6-518.8 pg/mL)	1.64 (1.11–2.75)*	1.51 (1.04–2.20)*
Tertile 3 (> 518.8 pg/mL)	2.60 (1.59–4.24)^‡^	2.34 (1.32–4.16)^‡^

The adjusted model included age, sex, hypertension, current smoking, systolic blood pressure, creatinine, low-density lipoprotein cholesterol, glycosylated hemoglobin, high sensitivity C-reactive protein, and baseline statin use

*CI* confidence interval, *DM* diabetes mellitus, *HR* hazard ratio, *LgNT-proBNP* log-transformed NT-proBNP, *MACEs* major adverse cardiovascular events, *NT-proBNP* N-terminal pro-B-type natriuretic peptide, *Pre-DM* prediabetes mellitus

^a^In the overall population, the adjusted model included the above variables plus DM

*p < 0.05, ^†^*p* < 0.01; ^‡^*p* < 0.001

Finally, we assessed whether the evaluation of NT-proBNP levels in addition to established coronary risk factors could improve risk stratification for MACEs in patients with CCS and pre-DM or DM under secondary prevention therapy in the real world. As presented in Table 3, adding NT-proBNP to traditional risk factors showed a significant improvement of the risk prediction for MACEs, with the C-index rising from 0.666 to 0.702 in patients with pre-DM (*p* = 0.018) and from 0.676 to 0.711 in patients with DM (*p* = 0.020).

**Table 3:** C-statistic of NT-proBNP for predicting major adverse cardiovascular events in patients with pre-DM or DM

	C-statistic (95% CI)	ΔC-statistic (95% CI)	*p* value
Pre-DM			
Original model	0.666 (0.615–0.718)		
Original model + NT-proBNP	0.702 (0.650–0.754)	0.035 (0.012–0.071)	0.018
DM			
Original model	0.676 (0.626–0.726)		
Original model + NT-proBNP	0.711 (0.661–0.761)	0.035 (0.005–0.063)	0.020

Original model included age, sex, hypertension, current smoking, systolic blood pressure, creatinine, low-density lipoprotein cholesterol, glycosylated hemoglobin, high sensitivity C-reactive protein, and baseline statin use

*CI* confidence interval, *DM* diabetes mellitus, *NT-proBNP* N-terminal pro-B-type natriuretic peptide, *Pre-DM* prediabetes mellitus

## Discussion

Over the years, the prognostic significance of NT-proBNP in patients with HF has been well established. Amazingly, the predictive role of this biomarker in a broader spectrum of CVDs has been confirmed in recent years. This study is the first to evaluate NT-proBNP as a prognostic parameter in a real-life cohort with CCS and normal LVSF according to different glucose status. Interestingly, our data showed that in prediabetic population, patients in tertile 2 and tertile 3 of NT-proBNP had 2.39-fold and 2.56-fold increases of the risk for MACEs respectively, compared with subjects in the lowest tertile. Additionally, per 1-SD increase of LgNT- proBNP was associated with a 61% increase of the risk of CVEs. Moreover, adding NT-proBNP to the model of established risk factors significantly improved the risk prediction for MACEs. Besides, we observed similar associations between NT-proBNP levels and worse cardiovascular outcomes in CCS patients with normal LVSF and DM, but not in those with normoglycemia. Thus, the present study suggested a prognostic utility of NT-proBNP in statin-treated CCS patients with normal LVSF and dysglycemia, supplying novel information and evidence for the clinical application of this biomarker.

It is worth mentioning that our study has focused more on the predictive role of NT-proBNP in patients with CCS and dysglycemia, especially in those with pre-DM. As well known, pre-DM, defined as impaired fasting glucose (IFG), impaired glucose tolerance (IGT), or raised HbA1c, reflects the natural history of progression from normoglycemia to DM. It has been reported that about 5–10% of individuals with pre-DM will become diabetic annually [23]. According to an ADA expert panel, up to 70% of people with pre-DM will eventually develop DM [23]. In a Chinese DM prevention trial, the 20-year cumulative incidence of DM was even higher (> 90%) among subjects with IGT [24]. In recent years, the prevalence of DM and pre-DM has been increasing worldwide and experts anticipate that more than 600 million individuals would develop DM by 2045, with around the same number developing pre-DM [18]. Moreover, similar to DM, pre-DM has been suggested to be associated with increased risk of CAD, composite CVEs, stroke, and all-cause mortality [21]. Thus, in line with previous studies [25, 26], there was a high percentage of pre-DM and DM in constituent ratio of our cohort who had angiography-proved CCS. As stated in the 2019 ESC guidelines, the elevated risk of CAD starts at glucose levels below the cut-off point for DM (< 7 mmol/L) and increases with increasing glucose levels [18]. Subjects with a FPG range from 5.6 to < 6.1 mmol/L have a 1.11-fold (95% CI: 1.04–1.18) and those with a FPG range from 6.1 to < 7 mmol/L have a 1.17-fold (95 % CI: 1.08–1.26) higher risk of developing CAD [27]. A recent meta-analysis showed that individuals with IFG, IGT, or raised HbA1c levels (5.7-6.4%) had a 13%, 30%, and 25% increase of the risk for composite CVEs respectively, compared to those with normoglycemia. Additionally, based on the data from 18 studies, IFG was associated with a 1.06 to 1.17-fold (95% CI: 1.01–1.11) increased risk of stroke, while IGT was associated with a 1.20-fold (95% CI: 1.0-1.45) increased risk of stroke after multivariate adjustment [21]. Similarly, in the present study, besides the positive association between DM and the risk of MACEs among patients with CCS, pre-DM was also significantly associated with elevated risk of MACEs. Thus, pre-DM is gaining more and more attention nowadays. The risk stratification and clinical management of this population becomes increasingly urgent and necessary to make steps to improve prognosis.

Up to now, previous studies including ours have demonstrated a series of risk factors for predicting cardiovascular outcomes in subjects with pre-DM or DM. Besides traditional cardiovascular risk factors [19, 28], numerous novel parameters, including lipoprotein(a) [29], fibrinogen [30], free fatty acids [31], cystatin C [32, 33] and so on, have emerged as significant cardiovascular risk factors in subjects with dysglycemia. However, given the growing prevalence and the added cardiovascular burden of pre-DM and DM, new avenues for exploration of more valuable prognostic biomarkers in these conditions is of increasing interest in cardiovascular field.

NT-proBNP, an established biomarker for the diagnosis and prognosis of HF [3–6], has been regarded as the most important marker for the risk of cardiac diseases [2]. For instance, in patients with CCS, NT-proBNP has been demonstrated to be significantly associated with the risk of CVEs and all-cause death [16, 34]. Similarly, increasing evidence has suggested that the NT-proBNP level provides prognostic information in patients with ACS [14, 35, 36]. Additionally, in the stent era, NT-proBNP has been reported to be a strong predictor of MACEs and mortality in patients after primary or selective PCI [37, 38]. Meanwhile, a newly published study showed that higher NT-proBNP level before primary PCI was independently associated with poor myocardial reperfusion in patients with ST-elevation MI [39]. Moreover, recent studies indicated that NT-proBNP levels were significantly associated with cardiovascular outcomes and mortality in patients with DM as well [2, 4, 12–15]. However, to our knowledge, few of previous studies has evaluated the prognostic significance of NT-proBNP in diabetic patients combined with CCS. Moreover, when it comes to the pre-DM population, there has been only one primary prevention study investigating the association between NT-proBNP levels and cardiovascular risk in prediabetic individuals. In the present study, we revealed, for the first time, that adding NT-proBNP to the prediction model could provide additional prognostic information beyond the traditional risk factors in prediabetic patients with CCS and normal LVSF. Additionally, our findings supplied further evidence for the improvement in discriminative ability by the addition of NT-proBNP to the established risk factors in patients with DM and CCS. In consistent with previous studies [13, 14, 34, 40, 41] the statistically significant improvement of C-index by adding NT-proBNP was modest, but NT-proBNP was suggested to be superior to traditional risk factors for predicting cardiovascular events in prediabetic or diabetic patients with CCS. Based on the above findings, the measurement of NT-proBNP in patients with dysglycemia and CCS might be meaningful in clinical practice. However, in CCS patients with normoglycemia, we observed no significant associations between NT-proBNP levels and the risk of MACEs, which may need further studies with a large sample size of this population to confirm.

The mechanisms by which NT-proBNP manifests as such a strong predictor of CVEs in subjects without HF have not yet been fully understood. Although NT-proBNP is released by the ventricular myocardium as a counterregulatory response to increased stress on the wall, vasoconstriction, and sympathetic tone, it may also be associated with the regulation of numerous physiologic functions that control energy metabolism [12], myocardial ischemia due to CAD or other cardiac pathological conditions [42, 43], and the development of end-organ damage including left ventricular hypertrophy, peripheral arterial disease, and glomerulosclerosis [44]. In addition, the increase of NT-proBNP levels may reflect subclinical levels of ventricular dysfunction or diastolic dysfunction, vascular dysfunction, and the activation of the renin-angiotensin-aldosterone system as well [38]. All of them could bring about poor cardiovascular prognosis. Undoubtedly, the exact mechanisms underlying the relationships between NT-proBNP and MACEs in various populations warrants further research.

This study is limited by several facets. First, this is an observational study, we cannot conclude whether NT-proBNP is causally related to the development of MACEs. Second, we did not measure NT-proBNP dynamically, so it remains unclear whether repeated measurement of NT-proBNP can provide further incremental value for prediction of MACEs. Third, the follow-up time of the present study needed to be longer in order to better examine the prognostic value of NT-proBNP in the long-term outcomes.

## Conclusions

In conclusion, elevated NT-proBNP levels are independent predictors of MACEs in patients with CCS, with the prognostic value of which mainly existed in patients with pre-DM and DM. Among CCS patients with abnormal glucose metabolism, the addition of NT-proBNP to the original model incorporating traditional risk factors yielded a significant increment of predictive value. Further studies may be needed to confirm our novel findings.

## Supplementary Information

**Additional file 1:** **Table S1.** Characteristics of the subjects with or without MACEs. **Table S2.** The multivariate Cox regression analysis of MACEs in prediabetic or diabetic patients with CCS. **Figure S1. **The incidence rate of major adverse cardiovascular events in the study population. DM, diabetes mellitus; Pre-DM, prediabetes mellitus. *p < 0.05 compared with the normoglycemia group.

## Data Availability

The datasets used and analyzed during the current study are available from the corresponding author on reasonable request.

## Abbreviations

ACS: Acute coronary syndrome
ADA: American Diabetes Association
CAD: Coronary artery disease
CVD: Cardiovascular disease
CVE: Cardiovascular event
cTnI: Cardiac troponin I
CK: Creatine kinase
CABG: Coronary artery bypass grafting
DM: Diabetes mellitus
DBP: Diastolic blood pressure
FPG: Fasting blood glucose
HF: Heart failure
HDL-C: High-density lipoprotein cholesterol
HsCRP: High-sensitivity C-reactive protein
HbA1c: Glycosylated hemoglobin
IFG: Impaired fasting glucose
IGT: Impaired glucose tolerance
LVSF: Left-ventricular systolic function
LDL-C: Low-density lipoprotein cholesterol
MACE: Major adverse cardiovascular event
MI: Myocardial infarction
NT-proBNP: N-terminal pro-brain natriuretic peptide
Pre-DM: Prediabetes mellitus.
PCI: Percutaneous coronary intervention
CCS: Chronic coronary syndrome
SBP: Systolic blood pressure
