# Supplementary material for: Prognostic value of NT-proBNP in patients with chronic coronary syndrome and normal left ventricular systolic function according to glucose status: a prospective cohort study
Source: Cardiovasc Diabetol. 2021 Apr 22;20:84. doi: 10.1186/s12933-021-01271-0 (PMC8063320; doi:10.1186/s12933-021-01271-0)
Supplement: Supplementary file 1 — Additional file 1: Table S1. Characteristics of the subjects with or without MACEs. Table S2. The multivariate Cox regression analysis of MACEs in prediabetic or diabetic patients with CCS. Figure S1. The incidence rate of major adverse cardiovascular events in the study population. DM, diabetes mellitus; Pre-DM, prediabetes mellitus. *p < 0.05 compared with the normoglycemia group. [file 12933_2021_1271_MOESM1_ESM.docx]

**Revised CVDB-D-21-00085**

**Additional file 1**

**Table S1.** Characteristics of the subjects with or without MACEs

| Variable | Event  (n=569) | Non-event  (n=7493) | *p* value |
| --- | --- | --- | --- |
| Age, years | 62.2±9.9 | 57.6±10.2 | <0.001 |
| Male, n (%) | 401 (70.5) | 5383 (71.8) | 0.465 |
| Hypertension, n (%) | 410 (72.0) | 4664 (62.2) | <0.001 |
| DM, n (%) | 257 (45.2) | 2695 (36.0) | 0.001 |
| Current smokers, n (%) | 219 (38.5) | 3162 (42.2) | 0.136 |
| Family history of CAD, n(%) | 73 (12.8) | 1062 (14.2) | 0.449 |
| Prior PCI, n (%) | 158 (27.8) | 1985 (26.5) | 0.578 |
| Prior CABG, n (%) | 30 (5.3) | 176 (2.3) | <0.001 |
| Prior MI, n (%) | 211 (37.1) | 2032 (27.1) | <0.001 |
| BMI, kg/m^2^ | 25.73±3.22 | 25.89±3.17 | 0.352 |
| SBP, mmHg | 129±18 | 127±17 | 0.018 |
| DBP, mmHg | 77±11 | 78±11 | 0.030 |
| LVEF, % | 62.23±8.10 | 64.21±6.78 | <0.001 |
| NT-proBNP, pg/mL | 556.2 (366.1-957.3) | 301.5 (57.6-559.0) | <0.001 |
| FPG, mmol/L | 5.97±1.92 | 5.86±1.76 | 0.244 |
| HbA1c, % | 6.58±1.20 | 6.31±1.09 | <0.001 |
| TC, mmol/L | 4.17±1.20 | 4.12±1.16 | 0.430 |
| HDL-C, mmol/L | 1.05±0.29 | 1.06±0.29 | 0.622 |
| LDL-C, mmol/L | 2.49±1.02 | 2.49±1.00 | 0.942 |
| TG, mmol/L | 1.53 (1.07-2.13) | 1.49 (1.10-2.08) | 0.656 |
| Creatinine, umol/L | 80.31±18.91 | 77.67±18.16 | 0.008 |
| HsCRP, mg/L | 1.79 (0.94-3.79) | 1.33 (0.73-2.74) | <0.001 |
| Baseline Medications |  |  |  |
| Aspirin, n (%) | 426 (74.9) | 5627 (75.1) | 0.289 |
| Statins, n (%) | 411 (72.2) | 5784 (77.2) | 0.007 |
| ACEI/ARB, n (%) | 106 (18.3) | 1624 (21.7) | 0.099 |
| β-blockers, n (%) | 237 (41.7) | 3127 (41.7) | 0.987 |
| CCB, n (%) | 106 (18.6) | 1491 (19.9) | 0.330 |
| Follow-up Medications |  |  |  |
| Aspirin, n (%) | 562 (98.7) | 7429 (99.1) | 0.672 |
| Statins, n (%) | 559 (98.2) | 7248 (96.7) | 0.092 |
| ACEI/ARB, n (%) | 323 (56.8) | 3499 (46.7) | <0.001 |
| β-blockers, n (%) | 453 (79.7) | 5822 (77.7) | 0.354 |
| CCB, n (%) | 228 (40.1) | 2891 (38.6) | 0.269 |

Continuous values are summarized as mean ± SD, median (interquartile range) and categorical variables as percentage. ACEI, angiotensin converting enzyme inhibitors; ARB, angiotensin receptor blockers; BMI, body mass index; CCB, calcium channel blockers; CABG, coronary artery bypass grafting; DM, diabetes mellitus; DBP, diastolic blood pressure; FPG, fasting plasma glucose; HbA1c, [glycosylated](javascript:void(0);) [hemoglobin](javascript:void(0);); HDL-C, high-density lipoprotein cholesterol; HsCRP, high sensitivity C-reactive protein; LVEF, left ventricular ejection fraction; LDL-C, low-density lipoprotein cholesterol; MACEs, major adverse cardiovascular events; MI, myocardial infarction; NT-proBNP, N-terminal pro-B-type natriuretic peptide; PCI, percutaneous coronary intervention; SBP, systolic blood pressure; TC, total cholesterol; TG, triglyceride.

**Table S2.** The multivariate Cox regression analysis of MACEs in prediabetic or diabetic patients with CCS

| Variable | Pre-DM | | DM | |
| --- | --- | --- | --- | --- |
|  | HR (95%CI) | *p* value | HR (95%CI) | *p* value |
| Age^*^ | 1.17 (0.94-1.46) | 0.149 | 1.38 (1.11-1.71) | 0.003 |
| Male | 0.92 (0.56-1.51) | 0.743 | 1.14 (0.70-1.87) | 0.597 |
| Hypertension | 1.19 (0.76-1.86) | 0.459 | 1.65 (0.97-2.80) | 0.063 |
| Current smoking | 1.25 (0.78-2.00) | 0.361 | 1.11 (0.71-1.73) | 0.653 |
| SBP^*^ | 1.05 (0.86-1.29) | 0.612 | 1.19 (0.98-1.44) | 0.079 |
| HbA1c^*^ | 1.20 (0.95-1.51) | 0.121 | 1.08 (0.89-1.31) | 0.432 |
| LgNT-proBNP^*^ | 1.61 (1.23-2.12) | <0.001 | 1.69 (1.30-2.21) | <0.001 |
| LDL-C^*^ | 1.08 (0.88-1.32) | 0.488 | 0.91 (0.73-1.15) | 0.445 |
| Creatinine^*^ | 1.10 (0.97-1.26) | 0.146 | 1.11 (0.95-1.30) | 0.180 |
| LghsCRP^*^ | 1.24 (1.00-1.54) | 0.051 | 1.09 (0.89-1.33) | 0.416 |
| Baseline statin use | 0.70 (0.45-1.07) | 0.099 | 0.87 (0.57-1.34) | 0.530 |

CCS, chronic coronary syndrome; DM, diabetes mellitus; HbA1c, [glycosylated](javascript:void(0);) [hemoglobin](javascript:void(0);); LgNT-proBNP, log-transformed N-terminal pro-B-type natriuretic peptide; LDL-C, low-density lipoprotein cholesterol; LgHsCRP, log-transformed high sensitivity C-reactive protein; MACEs, major adverse cardiovascular events; Pre-DM, prediabetes mellitus; SBP, systolic blood pressure.

^*^per 1-SD increase.

**Figure S1. The incidence rate of major adverse cardiovascular events in the study population.** DM, diabetes mellitus; Pre-DM, prediabetes mellitus.

^*^*p*<0.05 compared with the normoglycemia group.
